# Chronic Hepatitis B Relapse Rates after Cessation of Tenofovir Alafenamide and Entecavir Therapy

**DOI:** 10.3390/biomedicines11030752

**Published:** 2023-03-01

**Authors:** Chih-Wen Huang, Chen-Ta Yang, Pei-Yuan Su, Yang-Yuan Chen, Siou-Ping Huang, Hsu-Heng Yen

**Affiliations:** 1 Division of Gastroenterology, Department of Internal Medicine, Changhua Christian Hospital, Changhua 500, Taiwan; 2 College of Medicine, National Chung Hsing University, Taichung 400, Taiwan

**Keywords:** chronic hepatitis B, entecavir, tenofovir alafenamide, finite therapy, clinical relapse

## Abstract

Chronic hepatitis B (CHB) relapse occurs after the cessation of nucleos(t)ide analogues (NUC) therapy due to the waning of viral suppression. Few studies have investigated the viral relapse rate and clinical relapse rate after tenofovir alafenamide (TAF) therapy. We compared the CHB relapse rate between TAF and entecavir therapy. We enrolled patients with chronic hepatitis B who underwent TAF or entecavir therapy. NUC therapy was terminated after HBeAg loss for 1 year in HBeAg-positive patients and after undetectable serum HBV DNA on three separate tests each >6 months apart in HBeAg-negative patients. After cessation of NUC therapy, we followed alanine aminotransferase (ALT) levels at 12, 24, and 48 weeks. Serum HBV DNA levels were checked if patients showed a two-fold elevation from the upper limit of normal ALT levels (41 IU/mL). Clinical relapse (CR) was defined as a two-fold elevation in ALT levels and HBV DNA levels > 2000 IU/mL. We then investigated the CR rate of HBV after cessation of TAF and entecavir therapy at 12, 24, and 48 weeks. Of the 117 patients enrolled, 78 were in the entecavir group and 39 were in the TAF group. At 12 weeks after cessation of NUC therapy, no patients had HBV CR in the entecavir group. However, three patients (CR cumulative rate 7.9%) had CR in the TAF group. At 24 weeks, the CR cumulative rate in the entecavir and TAF groups were 1.3% and 13.2%, respectively (*p* < 0.05). At 48 weeks, the CR cumulative rates were 9.2% and 24.2%, respectively (*p* = 0.055). Patients in the TAF group had a higher cumulative rate of CR than those in the entecavir group (log-rank *p* = 0.023). Furthermore, patients in the TAF group had earlier CR times than those in the entecavir group, especially in the first 24 weeks after cessation of therapies (*p* < 0.05). The cessation of TAF therapy had significantly earlier and higher CR rates than that of entecavir therapy. Close monitoring of liver function and HBV DNA levels may be necessary, especially within 24 weeks after cessation of TAF therapy.

## 1. Introduction

The prevalence of hepatitis B virus (HBV) infection decreases gradually after vaccination. However, chronic HBV infection still plays a key role in the progression to cirrhosis and hepatocellular cell carcinoma (HCC). Nucleos(t)ide analogues (NUC) therapy can suppress HBV and reduce the possibility of cirrhosis and HCC [1]. Because of the excellent suppression of viral replication ability and barriers to virus resistance, NUC therapies such as entecavir, tenofovir disoproxil fumarate (TDF), and tenofovir alafenamide (TAF) are recommended as first-line therapy for chronic HBV infections [2]. Although NUC therapy can suppress HBV replication and reduce hepatocellular inflammation, it still cannot completely eradicate HBV [1]. HBsAg seroconversion is rare after long-term NUC therapy [3], and HBV relapse could occur after cessation of NUC therapy due to the waning of viral suppression [4,5,6,7,8]. However, the side effects of long-term NUC therapy need to be considered, such as entecavir-associated lactic acidosis, TDF-associated renal injury and osteoporosis [9,10], medication adherence, and financial issues. Furthermore, a study found that 25 out of 33 HBeAg-negative patients (76%) had clinical relapse (CR) after four or five years of adefovir dipivoxil treatment, while 39% of patients had hepatitis B surface antigen (HBsAg) loss. In these patients, discontinued antiviral therapy was the independent factor to achieve HBsAg loss [11]. A randomized controlled trial (FINITE trial) found that four out of 21 patients (19%) had HBsAg loss three years after cessation of TDF therapy, whereas no patients had HBsAg loss in the continued TDF treatment group [12]. These studies demonstrate that finite therapy achieved the functional cure of HBV more than continued treatment in HBeAg-negative patients. However, HBV relapse after cessation of NUC therapy may induce hepatic failure and even death. Finite NUC therapy still needs further evaluation to assess its risks and benefits. With regard to cessation of NUC therapy, the guidelines of the American Association for the Study of Liver Diseases, the European Association for the Study of the Liver, and the Asian Pacific Association for the Study of the Liver (APASL) suggest to discontinue NUC therapy after HBeAg loss and consolidation therapy >12 months in HBeAg-positive CHB patients. The APASL guidelines also suggest to discontinue NUC therapy in HBeAg-negative CHB patients after treatment for at least two years with undetectable HBV DNA documented on three separate occasions six months apart [2,13,14]. Recently, TAF, a prodrug of tenofovir, was approved for the treatment of chronic HBV infection. Compared with TDF, TAF therapy produced a lower systemic concentration and higher intracellular tenofovir concentration [15]. Our previous study showed that switching to TAF from TDF had good antiviral effectiveness [7]. To our knowledge, few studies have investigated the CR rate in these switched patients after TAF therapy. Therefore, we compared the CR rate between TAF and entecavir therapies 48 weeks after cessation of their antiviral therapy.

## 2. Materials and Methods

### 2.1. Materials

This was an observational, retrospective, single-center study. We enrolled adult patients with chronic hepatitis B who received antiviral therapy at the Changhua Christian Hospital from January 2015 to September 2021. The inclusion criteria comprised chronic HBV-infected patients who received TAF or entecavir therapy for at least six months at the Changhua Christian Hospital in accordance with the Taiwanese National Health Insurance reimbursement guidelines. Based on these guidelines, NUCs could be prescribed in Taiwan if an HBeAg-positive patient was HBsAg-positive for more than half a year, had a two- to five-fold increase from the upper limit of the normal alanine aminotransferase (ALT) level (41 IU/mL), and an HBV DNA level > 20,000 IU/mL; if HBeAg-negative patients were HBsAg-positive for more than half a year, had a two- to five-fold increase from the upper limit of normal ALT levels, and HBV DNA levels greater than 2000 IU/mL; if HBsAg-positive patients had a five-fold elevation from the upper limit of normal ALT levels; and if HBsAg-positive patients had liver decompensation (prothrombin time above 3 s and total bilirubin above 2 mg/dL). NUC therapy should be terminated after HBeAg loss for 1 year in HBeAg-positive patients, and after undetectable serum HBV DNA levels on three separate occasions more than 6 months apart in HBeAg-negative patients.

The exclusion criteria were patients with HCC, cirrhosis, alcoholic liver disease, hepatitis C virus infection, evidence of autoimmune hepatitis, and immunosuppressive therapy during NUC treatments. A total of 139 patients were enrolled according to the inclusion and exclusion criteria. 

**Figure 1:**
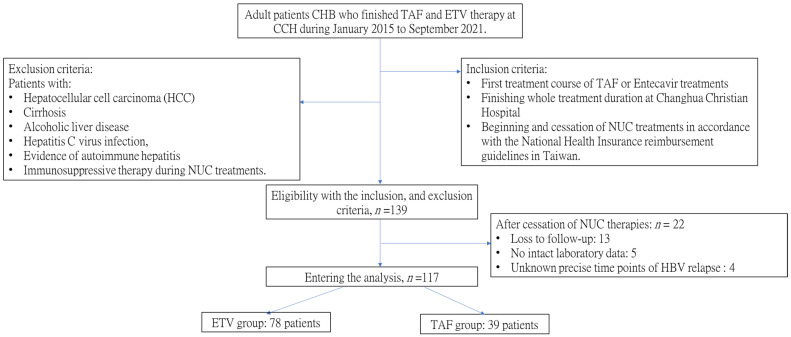
Patient disposition during the study. CHB, chronic hepatitis B; TAF, tenofovir alafenamide; ETV, entecavir; CCH, Changhua Christian Hospital.

Of the 139 patients, 13 were lost to follow-up; 5 had no intact laboratory data after cessation of NUC treatment; and 4 had no regular follow-up and experienced HBV relapse at unknown time points after cessation of NUC. Ultimately, 117 patients were enrolled for analysis. Of these, 16 (14%) were HBeAg-positive while 101 (86%) were HBeAg-negative. Furthermore, 78 patients were in the entecavir group, whereas 39 were in the TAF group. Figure 1 presents the flowchart of the patient enrollment process.

### 2.2. Follow-Up Strategy after Cessation of NUC Therapy and Definition of Clinical Relapse

After cessation of NUC therapy, we monitored ALT at 12, 24, and 48 weeks. The serum HBV DNA level was measured if a patient showed a two-fold elevation from the upper limit of normal ALT levels (41 IU/mL). Clinical relapse (CR) was defined as two-fold elevation in ALT levels and HBV DNA levels > 2000 IU/mL. Then, the CR rate of HBV was compared between TAF and entecavir at 12, 24, and 48 weeks. If the patients had CR, the total bilirubin and prothrombin time would be checked to determine liver decompensation.

The study was conducted in accordance with the Declaration of Helsinki and approved by the Institutional Review Board of the Changhua Christian Hospital Protocol code 230,107, date of approval 12 January 2023.

### 2.3. Statistical Analyses

Data are expressed as *n* (%), median (interquartile range), or mean ± standard deviation. The independent sample t-test was used to analyze the relationship between TDF therapy duration and CR in the TAF group. The distribution of continuous variables was analyzed using the one-sample Kolmogorov–Smirnov test. Categorical variables were compared using the *χ^2^* test; continuous variables were compared using Student’s t-test or the Mann–Whitney U-test, as appropriate. The entry date was the day of end of treatment (EOT). The time at risk as a continuous variable was measured from the entry date until CR or the last date of normal ALT inspection, whichever came first. The Kaplan–Meier method with a log-rank test was used to analyze the cumulative rate of CR. R software for Windows, version 4.0.5, and the “survival” (https://cran.r-project.org/web/packages/survival/index.html, accessed on 21 January 2023) and “survminer” (https://cran.r-project.org/web/packages/survminer/index.html, accessed on 21 January 2023) packages were used to generate cumulative incidence plots. Statistical analysis was performed using IBM SPSS Statistics for Windows, version 22.0 (IBM Corp., Armonk, NY, USA). A *p*-value less than 0.05 was considered statistically significant.

## 3. Results

### 3.1. General Characteristics of the Study Population

Overall, 117 patients were enrolled for analysis. The clinical features of the entecavir and TAF groups are shown in Table 1. Seventy-eight patients comprised the entecavir group, whereas 39 patients comprised the TAF group. Twelve and four patients were HBeAg-positive in the entecavir and TAF groups, respectively. No significant differences were found in age, sex, baseline ALT level, total bilirubin level, and serum HBV DNA level between the two groups. At EOT, the median HBsAg levels were 780.69 IU/mL (208.1–1992.74 IU/mL) and 560.15 IU/mL (317.82–1109.89 IU/mL) for the entecavir and TAF groups (*p* = 0.524), respectively. The treatment durations were all 3 years in both groups. In the TAF group, all patients underwent first treatment with TDF and then shifted to TAF. The treatment duration of TAF ranged from 0.87 to 1.83 years.

### 3.2. Relapse Pattern of Entecavir and TAF

**Figure 2:**
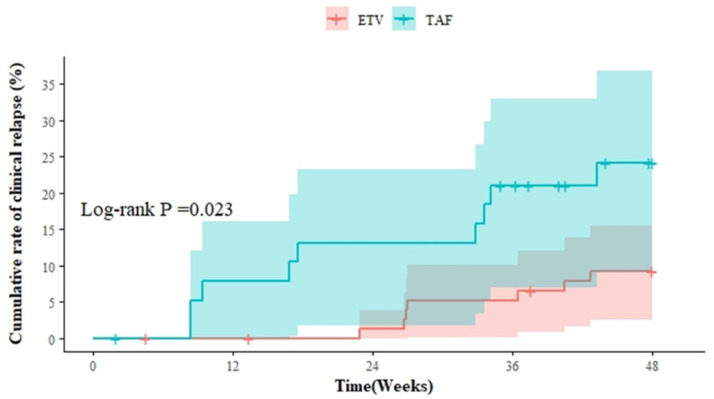
Comparison of HBV clinical relapse after cessation of either entecavir or TAF. The Kaplan–Meier method with a log-rank test was used to analyze the cumulative rate of clinical relapse. R software and the “survival” (https://cran.r-project.org/web/packages/survival/index.html, accessed on 21 January 2023) and “survminer” (https://cran.r-project.org/web/packages/survminer/index.html, accessed on 21 January 2023) packages were used to generate cumulative incidence plots. ETV, entecavir; TAF, tenofovir alafenamide.

Sixteen patients experienced HBV CR, seven in the entecavir group and nine in the TAF group. Of the 78 patients in the entecavir group, seven (9%) experienced CR. The cumulative rates of CR at 24 and 48 weeks were 1.3% and 9.2%, respectively. No CR was observed at 12 weeks. Of the 39 patients in the TAF group, nine experienced CR (23%). The cumulative rates of CR at 12, 24, and 48 weeks were 7.9%, 13.2%, and 24.2%, respectively. Forty-eight weeks after cessation of NUC therapy, patients had a higher cumulative rate of CR in the TAF group than in the entecavir group (log-rank *p* = 0.023) (Figure 2). Furthermore, patients in the TAF group had earlier times of CR than in the entecavir group, especially during the first 24 weeks after cessation of therapies (*p* < 0.05) (Table 2).

All patients in the TAF group were treated with TDF first and then shifted to TAF. The mean TDF therapy duration was 1.99 ± 0.56 years in patients with HBV CR and 1.58 ± 0.59 years in patients without CR. However, the TDF therapy duration was not associated with CR (*p* = 0.077) (Table 3).

### 3.3. Incidence of Liver Decompensation after Cessation of NUC Therapies

After cessation of TAF and entecavir therapies, three patients (2.6%), two in the entecavir group (2.6%) and one in the TAF group (2.6%), developed hyperbilirubinemia. The peak levels of total bilirubin elevation were 2.2, 4.5, and 5.5 mg/dL. One of them also had coagulopathy with prolongation of international normalized ratio (INR: 1.53). The times to liver decompensation were 41.9 weeks (patient in the TAF group), 22.6 weeks (patient in the entecavir group), and 44.7 weeks (patient in the entecavir group) after cessation of NUC therapies. The mean time to liver decompensation was 36.4 weeks. 

## 4. Discussion

Although finite NUC therapy may minimize the concerns of indefinite long-term therapy and much increased HBsAg loss in HBeAg-negative patients [16], CR may occur, cause hepatitis flare, and sometimes deteriorate to hepatic decompensation and even death. Kuo et al. [8] reported that the 2-year cumulative rate of CR was 37.6% in HBeAg-positive patients, whereas the 3-year cumulative rate of CR was 51.6% in HBeAg-negative patients after TDF or entecavir treatment. The TDF group tended to have a more severe CR rate than the entecavir group. Another retrospective study in Taiwan found that, at 3 months after cessation of NUC treatments, patients had significantly higher rates of CR with TDF than with entecavir treatment. However, no significant difference was observed in the CR rate at 12 months [7]. CR was also observed earlier after the cessation of TDF than after the cessation of entecavir in HBeAg-negative patients [6]. As a prodrug of tenofovir, TAF has the same mechanism of action as TDF and has been approved for the treatment of chronic hepatitis B infection as a replacement of long-term TDF therapy. In two double-blind, randomized trials, TAF was as effective as TDF, with improved renal and bone safety, 2 years after the initiation of treatment [17]. Despite the theoretical efficiency of TAF and TDF, information on the CR rate after cessation of TAF is more limited than that on other NUC treatments. In the present study, we found that patients in the TAF group had significantly earlier and higher CR rates than those in the entecavir group. Most patients developed CR within 6 months after the cessation of TAF therapy. Therefore, close monitoring in the first 6 months after cessation of TAF therapy is recommended. 

Only 16 HBeAg-positive patients were included in our study. Of these, only two had CR, one in each treatment group. Hence, we did not discuss the role of HBeAg in CR after the cessation of NUC treatments because of the small population of HBeAg-positive patients. Compared with previous studies [7,8], our study reported lower CR rates regardless of treatment group. A previous study showed that HBsAg levels at EOT, CTLA4 (rs231775), and non-GG genotype significantly predicted CR. Patients with an HBsAg level above 200 IU/mL had a significantly higher risk of CR. Baseline HBeAg-positive status and HBsAg levels at EOT significantly predicted sustained clinical response [7]. The median HBsAg level at EOT in our study population was 750 IU/mL. The HBeAg-positive patients only accounted for 13.4% of our study population. However, we did not investigate the HBV genotype in our study. It may explain the lower CR rate we observed. 

This study was the first to compare the CR rate after treatment cessation between TAF and entecavir therapies. Compared with those reported in previous studies, TAF was similar to TDF and had earlier and higher CR rates than entecavir therapy, especially within the first 6 months after cessation. 

However, because TAF was included in the Taiwanese National Health Insurance in May 2019, the patients in the TAF group underwent TDF treatment first and were subsequently shifted to TAF after May 2019. TAF therapy lasted from 0.87 to 1.83 years. Hence, the evaluation of the sustained clinical response to TAF in this study may be relevant. The mean TDF therapy duration in patients with HBV CR was 1.99 years, whereas it was 1.58 years in patients without CR. However, the duration of TDF therapy was not associated with CR (*p* = 0.077). 

We followed the National Health Insurance reimbursement guidelines in Taiwan as the criteria for EOT. Only three patients (2.6%) had liver decompensation after the cessation of NUC therapies. Two patients developed hyperbilirubinemia (total bilirubin above 2 mg/dL), while one patient had hyperbilirubinemia and prolongation of prothrombin time with INR above 1.5 after the cessation of NUC therapy. Of the three patients, two in the entecavir group (2.6%) experienced liver decompensation 22.6 weeks and 44.7 weeks after cessation of entecavir. One patient in the TAF group underwent liver decompensation 41.9 weeks after cessation of TAF therapy. Hyperbilirubinemia and coagulopathy were remedied after retreatment with NUC therapy without changing the original NUC. The patients with liver decompensation after therapy cessation had higher baseline total bilirubin or ALT levels before NUC therapy. Unfortunately, these three patients have no complete laboratory data for analysis at EOT. The baseline ALT and bilirubin levels may be independent factors for predicting the incidence of liver decompensation after cessation of NUC therapy. However, more studies are needed to confirm this. No deaths or liver transplantations due to acute hepatitis flare-ups occurred after the cessation of therapies in our study. Thus, the finite NUC therapies using entecavir and TAF are feasible and reasonably safe. 

A systematic review and meta-analysis reported that severe hepatitis flares or decompensation occur in 1.21% of cases, and hepatitis flare-related death or liver transplantation occur in 0.37% of cases, with a mean follow-up duration of over 12 months [18]. However, this systematic review only included NUC therapies with entecavir and TDF. Liver decompensation after cessation of TAF therapy was 2.6% in our study. Compared with the previous study [18], our study had a higher liver decompensation rate. The reason was unclear, but the small population size may be the main cause. 

Our study has some limitations. First, it was relatively small, with just 117 patients enrolled. A lower CR rate was observed in our study, and events of CR in HBeAg-positive patients were few; thus, we did not further investigate the influence of HBeAg on the CR rate. Second, we did not investigate the genotypes of our chronic hepatitis B patients, which may influence the CR rate and explain the lower CR rate we observed. Third, this was a retrospective study of real-world data and was subject to the rules of National Health Insurance reimbursement. Hence, we checked the serum HBV DNA levels only if the patients showed a two-fold elevation from the upper limit of normal ALT levels (41 IU/mL). Hence, we did not compare the virological relapse (VR) rate between the TAF and entecavir groups. In previous studies, some patients experienced VR without CR [7,8], but the reason was unclear. Furthermore, the VR-only patients developed VR significantly later than patients who developed both VR and CR [7]. These patients had a superior ability of sustaining viral suppression after cessation of NUC therapy. The clinical implications of VR without CR still need further investigation.

## 5. Conclusions

Our study found different relapse patterns for TAF and entecavir therapies. Although a small proportion (2.6%) of patients experienced liver decompensation, no death or liver transplantation due to acute hepatitis occurred after the cessation of NUC therapy. The cessation of TAF therapy had significantly earlier and higher CR rates than that of entecavir therapy. Close monitoring of liver function and HBV DNA levels may be necessary after cessation.

## Figures and Tables

**Table 1:** Clinical features of the study population.

Characteristic	All Patients (*n* = 117)	Entecavir (*n* = 78)	TAF (*n* = 39)	*p*-Value
Gender (Male), *n* (%)	84 (71.8)	59 (75.6)	25 (64.1)	0.191
Age (years), mean ± SD	52.1 ± 12.2	52.9 ± 12.3	50.5 ± 11.9	0.325
Baseline ALT, U/L, median (IQR)	176 (107–609)	205 (105–666)	152 (112–545)	0.824
Bilirubin-T, mg/dL, median (IQR) ^a.^	1.25 (0.95–2.44)	1.26 (0.97–2.37)	1.25 (0.74–2.56)	0.792
HBeAg interpretation (Reactive), *n* (%)	16 (13.7)	12 (15.4)	4 (10.3)	0.447
Baseline Hepatitis B DNA, log IU/mL, median (IQR)	6.73 (5.59–7.62)	6.85 (5.71–7.77)	6.36 (5.42–7.54)	0.324
EOT ALT, U/L, median (IQR) ^b.^	21 (17–35)	21 (16–31.5)	22 (17–43)	0.400
EOT Bilirubin-T (mg/dL), mean ± SD ^c.^	0.93 ± 0.45	1 ± 0.48	0.77 ± 0.34	0.202
EOT HBsAg, median (IQR) ^d.^	750.56 (239.47–1752.48)	780.69 (208.1–1992.74)	560.15 (317.82–1109.89)	0.524
Treatment duration (years), median (IQR)	3 (3–3)	3 (3–3)	3 (3–3)	0.764
TDF duration (years), median (IQR) ^e.^	1.54 (1.2–2.11)		1.54 (1.2–2.11)	-
ETV/TAF duration (years), median (IQR)	3 (1.83–3)	3 (3–3)	1.53 (0.87–1.83)	<0.001

Data are expressed as *n* (%), median (interquartile range), or mean ± standard deviation. Categorical variables were compared using the χ^2^ test; continuous variables were compared using Student’s *t*-test or the Mann–Whitney U-test, as appropriate. *p* < 0.05 indicated statistical significance. TAF, tenofovir alafenamide; SD, standard deviation; ALT, alanine aminotransferase; IQR, interquartile range; EOT, end of treatment; HBsAg, hepatitis B serum antigen; TDF, tenofovir disoproxil fumarate; ETV, entecavir; a. Baseline bilirubin-T data were missing for 5 and 15 patients in the entecavir and TAF groups, respectively; b. EOT ALT data were missing for 10 and 8 patients in the entecavir and TAF groups, respectively; c. EOT bilirubin-T data were missing for 59 and 30 patients in the entecavir and TAF groups, respectively; d. EOT HBsAg data were missing for 38 and 18 patients in the entecavir and TAF groups, respectively; e. TDF duration data were for patients in the TAF group.

**Table 2:** Clinical relapse status for patients after cessation of entecavir or TAF treatments.

Time after EOT	Entecavir %, (95% CI)	TAF %, (95% CI)	*p*-Value
12 weeks	0 (-)	7.9 (2.7–23.4)	-
24 weeks	1.3 (0.2–9.2)	13.2 (5.8–29.8)	0.036
48 weeks	9.2 (4.6–18.7)	24.2 (13.7–42.9)	0.055

EOT, end of treatment; CI, confidence interval; TAF, tenofovir alafenamide.

**Table 3:** Relationship between TDF duration and clinical relapse in the TAF group.

	Number	Mean	SD
**Patients with CR**	9	1.99	0.56
**Patients without CR**	10	1.58	0.59

F test: *p* = 0.645; *t*-test: *p* = 0.077; CR, clinical relapse; SD, standard deviations.

## Data Availability

The analyzed data is available upon reasonable request.
